# Fluctuation of Arabidopsis seed dormancy with relative humidity and temperature during dry storage

**DOI:** 10.1093/jxb/erv439

**Published:** 2015-10-01

**Authors:** Isabelle Basbouss-Serhal, Juliette Leymarie, Christophe Bailly

**Affiliations:** ^1^Sorbonne Universités, UPMC Univ Paris 06, UMR 7622, 75005 Paris, France; ^2^CNRS, UMR 7622, 75005 Paris, France

**Keywords:** After-ripening, ageing, *Arabidopsis thaliana*, germination, relative humidity, temperature.

## Abstract

The changes in germination potential of freshly harvested seeds of *Arabidopsis thaliana* stored in various combinations of temperature and relative humidity were investigated over 63 weeks of storage. Seeds of the wild type Col-0 and of two mutants displaying low and high levels of dormancy, *cat2-1* and *mtr4-1*, respectively, were stored at harvest in 24 different environments including a combination of eight relative humidities, from 1 to 85%, and four temperatures (10, 15, 20, and 25 °C). These mutations did not influence behaviour of seeds during storage. Primary dormant seeds did not germinate in darkness at 25 °C but acquired the potential to germinate at this temperature within 7 weeks when stored in relative humidities close to 50% across all temperatures. Sorption isotherms and Arrhenius plots demonstrated that the seed moisture content of 0.06g H_2_O/g dry weight was a critical value below which dormancy release was associated with reactions of negative activation energy and above which dormancy release increased with temperature. Longer storage times when relative humidity did not exceed 75–85% led to decreased germination at 25 °C, corresponding to the induction of secondary dormancy. Dormancy release and induction of secondary dormancy in the dry state were associated with induction or repression of key genes related to abscisic acid and gibberellins biosynthesis and signalling pathways. In high relative humidity, prolonged storage of seeds induced ageing and progressive loss of viability, but this was not related to the initial level of dormancy.

## Highlight

Temperature and relative humidity occurring during prolonged storage of freshly harvested *Arabidopsis* seeds control dormancy cycling and the expression of molecular regulators of this process.

## Introduction

Orthodox seeds are anhydrobiotes; they withstand severe desiccation at the end of their development on the mother plant and they can survive under very limited amounts of water in a resting state for years or decades. The desiccated state allows the cytoplasm to enter the highly viscous glassy state in which active metabolism is virtually not possible since it is prevented by low molecular mobility (Buitink and Leprince, 2008). Molecular diffusion and probability of chemical reactions are so decreased in the glassy state that they prevent enzymatic reactions (Fernández-Marín *et al.*, 2013). However, despite their resting state, orthodox seeds undergo major physiological changes during their storage, such as dormancy alleviation and loss of viability, but the underlying mechanisms of these processes remain largely unknown.

Seed dormancy is defined as an inability to germinate even though all the necessary environmental conditions for germination are apparently satisfied (Bewley, 1997). This is an adaptive trait that permits the novel generation of plants issued from seeds to develop in the favourable season. Dormancy determines the seasonal timing of germination and plays a major role in the dynamics of plant establishment in ecosystems (Baskin and Baskin, 1998). Primary dormancy is induced during seed maturation but for many species it decreases with time under dry storage through a process called after-ripening (Finch-Savage and Leubner-Metzger, 2006). Seed after-ripening depends on seed moisture content (MC) [i.e. relative humidity (RH) of storage] and temperature. It is generally favoured when seed MC falls within the range 5–18% fresh weight (fw) basis, which corresponds to the intermediate region of water sorption isotherms (Probert, 2000). Bazin *et al.* (2011) demonstrated that the rate of dormancy alleviation during dry after-ripening depended on the relationship between seed MC and temperature, and they proposed that there existed an optimal MC for dormancy release at each temperature. Seeds can also display secondary dormancy, which is induced after seed dispersal when the conditions required for completing germination are absent (Baskin and Baskin, 1998). For example, prolonged imbibition at low water potential or warm temperatures can induce secondary dormancy in the *Arabidopsis thaliana* ecotype Columbia (Donohue *et al.*, 2007; Auge *et al.*, 2015). Interestingly, secondary dormancy can be lost and re-introduced with season changes until environmental conditions become favourable for allowing germination (Finch-Savage and Leubner-Metzger, 2006). Secondary dormancy participates in the dormancy cycling, which is defined as the annual pattern of changing dormancy status in the natural environment, i.e. in the soil seed bank (Footitt *et al.*, 2015). In *Arabidopsis* it has been demonstrated that depth of dormancy of buried seeds was correlated with seasonal variation of soil temperature (Footitt *et al.*, 2011). Changes in physiological status of dry seeds in their environment are particularly important in an ecological context since they determine the seasonal timing of seed germination (Baskin and Baskin, 1998; Auge *et al.*, 2015). They are highly relevant within the context of global warming since reduction of moisture and increase in temperature as a result of climate change may modify the rate of seed dormancy alleviation and thus the features of plant regeneration in natural ecosystems (Walck *et al.*, 2011). This also questions how dry resting seeds would be able to sense the environmental signals necessary for determining time and place of seed germination (Huang *et al.*, 2015) if soil water limitation prevents their hydration after dispersal.

Mutants altered in dormancy induction or expression and studies related to the expression of the *Arabidopsis* genome following imbibition of mature seeds indicate that there are very large changes in genome expression that are associated with the transition from dormancy to germination (Holdsworth *et al.*, 2008). It is widely accepted that abscisic acid (ABA) and gibberellins (GAs) are the hormones playing essential but antagonistic roles in dormancy and germination, and the relative prevalence of their metabolism and signalling pathways regulates the ability to germinate (Finch-Savage and Leubner-Metzger, 2006). Many genes are involved in induction and maintenance of seed dormancy (see Graeber *et al.*, 2012). They include seed maturation regulators (Graeber *et al.*, 2012), genes regulating reactive oxygen species (ROS) homeostasis (Leymarie *et al.*, 2012), or specific genes of dormancy such as DELAY OF GERMINATION 1 (DOG1), which encodes a protein of unknown function (Bentsink *et al.*, 2006). The molecular mechanisms involved in seed dormancy release during dry after-ripening are less well known. Even though some changes in transcript abundance have been evidenced during dry after-ripening (Finch-Savage *et al.*, 2007), it has been demonstrated that active transcription was prevented in dry seeds (Meimoun *et al.*, 2014). Oracz *et al.* (2007), Bailly *et al.* (2008), and Bazin *et al.* (2011) showed that seed dormancy release in the dry state was associated with ROS accumulation, which in turn triggered specific oxidations of proteins and mRNA, thus modifying cell functioning during subsequent seed imbibition.

The other major modification occurring during prolonged seed dry storage is loss of viability, the ultimate consequence of seed ageing. Seed ageing is an irreversible process initiated at seed shed but its kinetics greatly depends upon RH and temperature during storage, initial seed vigour, genetic background, and growth environment of the seed (Ellis and Roberts, 1980; Walters, 1998). Interdependence of RH, i.e. seed MC, and temperature has been evidenced by Harrington (1973) who proposed three ‘rules of thumb’ regarding optimal seed storage. The first one, applicable for seeds with MC ranging from 5 to 14%, states that each 1% reduction of seed MC doubles seed longevity; the second one states that for each 5–6 °C decrease in storage temperature seed storage life is doubled; and the third one states that the arithmetic sum of RH and storage temperature should not exceed 100 for safe seed storage, or 120 as later reported (Bewley and Black, 1985; Copeland and McDonald, 1999). Similar biochemical and molecular changes are likely to occur during prolonged seed storage than during after-ripening, which suggests that there is a continuum for the chemical reactions taking place at the onset of seed dispersal through dormancy release, ageing, and loss of viability. Since enzymatic reactions are not possible in dry seeds, ROS accumulation and oxidative modifications of biomolecules have often been considered as one of the most important factors influencing seed ageing (Bailly *et al.*, 2004). Thus, Bailly *et al.* (2008) suggested that seed germination occurred when the seed ROS content was enclosed within an oxidative window allowing ROS signalling, i.e. dormancy alleviation, but not ROS damage, i.e. seed ageing.

The objective of the present study was to determine how the conditions of storage of *Arabidopsis thaliana* seeds at harvest influence the kinetics of seed dormancy release (after-ripening) and ageing. To address this question, dormant *Arabidopsis* seeds were stored within a large range of temperatures and RH and germination were regularly assessed for 1 year. This study was carried out with seeds of the *Arabidopsis* ecotype Columbia (Col-0) and with seeds from *mtr4-1* (exosome RNA helicase, Lange *et al.*, 2011) and *cat2-1* (catalase 2, Queval and Noctor, 2007) mutants. When germinated at 25 °C in darkness, freshly harvested Col-0 seeds display marked dormancy since their germination is largely inhibited (Leymarie *et al.*, 2012; Basbouss-Serhal *et al.*, 2015). Using this experimental system, Basbouss-Serhal *et al.* (2015) recently demonstrated that *Arabidopsis* seed dormancy was regulated by selective recruitment of mRNAS to polysomes. In contrast, dormant Col-0 seeds are able to germinate at 25 °C under continuous light, or after cold stratification, for example, but any treatment that alters the conditions required for germination is by definition altering dormancy (Finch-Savage and Leubner-Metzger, 2006). Thus, light being a dormancy release factor (Finch-Savage and Leubner-Metzger, 2006), it was considered that an appropriate evaluation of fluctuation of seed dormancy during storage had to be carried out by germinating seeds in darkness, and this was done in this study. Under these conditions, seeds of the mutant *cat2-1* are low dormant at harvest (Leymarie *et al.*, 2012), whereas seeds of *mtr4-1* are deep dormant (Basbouss-Serhal *et al*., unpublished data). The use of seeds from mutants was undertaken to determine if the genotype might influence the storage behaviour of the seeds and if initial dormancy level could modulate rate of dormancy alleviation and seed longevity. Water sorption isotherms and Arrhenius plots were provided to get insights on the mechanisms involved in release of primary dormancy. The changes in the abundance of transcripts related to ABA and GA metabolism and signalling during seed storage were also followed. They include *NCED3* (Nine-*Cis*-Epoxycarotenoid Dioxygenase 3), *NCED6* (Nine-*Cis*-Epoxycarotenoid Dioxygenase 6), and *NCED9* (Nine-*Cis*-Epoxycarotenoid Dioxygenase 9), coding for 9-*cis*-epoxycarotenoid dioxygenases involved in ABA biosynthesis (Tan *et al.*, 2003, Lefebvre *et al.*, 2006), and *ABI5* (ABA Insensitive 5), which codes for a member of the basic leucine zipper transcription factor family and is involved in ABA signalling during seed maturation and germination (Finkelstein and Lynch, 2000). *CYP707A2* codes for an ABA 8-hydroxylase and is involved in ABA catabolism (Millar *et al.*, 2006). *GA3ox1* (GA3 oxidase 1) and *Ga20ox4* (GA 20-oxidase 4) code for dioxygenases which catalyse the conversion of gibberellin precursors to their bioactive forms (Yamaguchi, 2008), while *Ga2ox2* (GA2 oxidase 2) is involved in gibberellin inactivation (Yamaguchi, 2008). *SLP2* (Subtilisin-Like serine Protease 2) is induced by gibberellins (Ogawa *et al.*, 2003). Finally, the expression of *DOG1*, a major regulator of seed dormancy (Bentsink *et al.*, 2006; Dekkers and Bentsink, 2015) was studied. Here, it is demonstrated that dormancy level of *Arabidopsis* seeds fluctuates during dry storage in a complex manner dependent upon seed MC and temperature of storage.

## Material and methods

### Plant material and after-ripening conditions

*Arabidopsis thaliana* seeds were grown and harvested dormant as described by Leymarie *et al.* (2012). Col-0 (Columbia) was used as the wild type. The *mtr4-1* mutant (GABI-048G02, provided by Dr Hervé Vaucheret, Institut Jean-Pierre Bourgin, INRA, Versailles, France) is characterized by a T-DNA insertion in the *MTR4* gene (AT1G59760) (Lange *et al.*, 2011). The *cat2-1* mutant (accession No. SALK_057998) is characterized by a T-DNA insertion in the *CAT2* gene (AT4G35090) in the third exon from the 5′ end (Queval and Noctor, 2007). After-ripening was performed by placing seeds in darkness at 10, 15, 20, and 25 °C over silica gel or saturated solutions of ZnCl_2_, LiCl, KAc, MgCl_2,_ CaCl_2,_ CaNO_3_, NaCl, and KCl, in tightly closed jars, thus giving RH values of approximately 1, 5.5, 14, 23.3, 36, 45, 56, 75, and 85%, respectively (Vertucci and Roos, 1993; Sun, 2004).

### Germination assays

Germination assays were performed at 15 °C and 25 °C in darkness by placing 100 seeds of each genotype in 9cm Petri dishes on a layer of cotton wool moistened with water or with a solution of gibberellic acid (10^–4^ M) dissolved in water. Ethylene treatment was carried out by placing open Petri dishes with seeds imbibed on water in a closed container containing 200 ppm ethylene. A seed was considered as germinated when the radicle protruded the envelopes. Germination was scored daily for 7 d and the results presented correspond to the mean of the germination percentages obtained for three replicates of 100 seeds ± standard deviation (SD).

### Tetrazolium test

After 48h of imbibition in water at room temperature, seeds were transferred to 1% (w/v) tetrazolium chloride (TZ) solution and incubated in darkness for a further 24h at 25 °C before staining evaluation (Delouche *et al.*, 1962). Results presented correspond to the percentage of seeds developing red staining in the presence of TZ solution and are the means of three replicates of 25 seeds ± SD.

### MC determination

Seeds (~15mg fw, in three replicates) were dried at 105 °C for 24h and dry weight was subsequently measured with a microbalance. Seed MC was determined as follows: fw – dw (dry weight)/dw. Results are expressed as g H_2_O g^–1^on a dry weight basis.

### RNA extraction and real-time quantitative RT-PCR

Thirty milligrams of seeds (three biological replicates) were ground in liquid nitrogen and total RNA was extracted by a hot phenol procedure according to Verwoerd *et al.* (1989). Reverse transcription was performed with 1 µg total RNA as described by Leymarie *et al.* (2012). The relative expressions were calculated according to Hellemans *et al.* (2007) with two reference genes, *Ubiquitin 5* and AT4G12590, and expressed in arbitrary units with a value of 100 to the dry dormant seeds after harvest, which was used as control sample for normalization (Pfaffl, 2001). Primer sequences are presented in Supplementary Table S1 (available at *JXB* online). The statistical analysis was performed with StatBox 6.40 software (Grimmer logiciels, France). Data were normally distributed and equal variance Newman–Keuls tests (*α*= 0.05) were performed after analysis of variance (ANOVA).

## Results

### Effect of RH and temperature on seed dormancy release

**Fig. 1.**
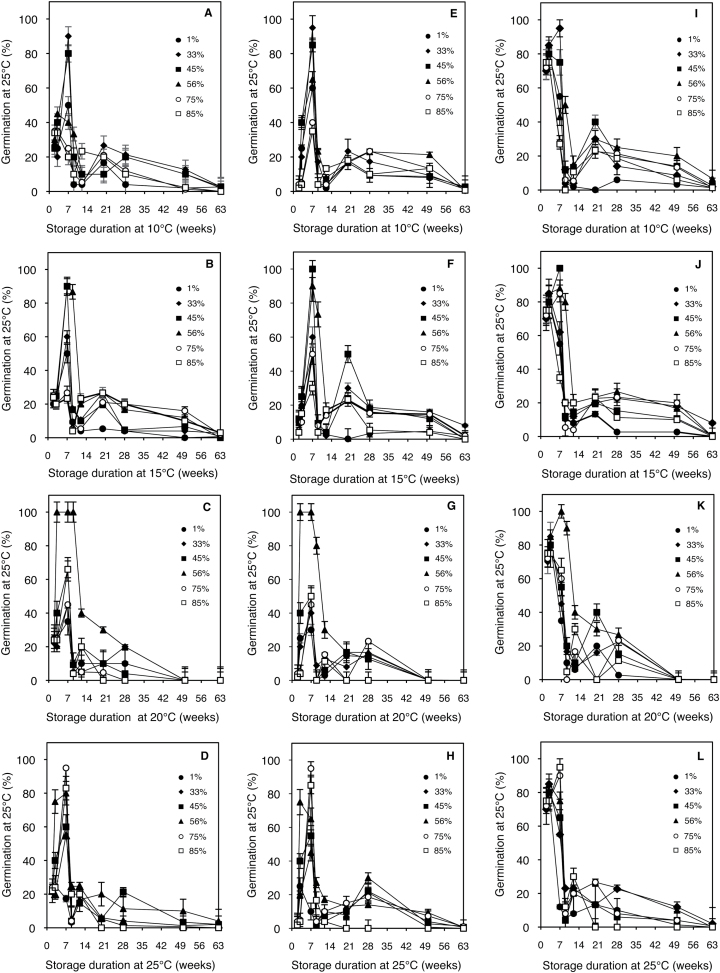
Seed germination percentages at 25 °C in darkness of wild type Col-0 (A, B, C, D), *mtr4-1* (E, F, G, H), and *cat2-1* (I, J, K, L) after different durations of storage at 10 °C (A, E, I), 15 °C (B, F, J), 20 °C (C, G, K), and 25 °C (D, H, L) under RHs of 1% (black circles), 33% (diamonds), 45% (black squares), 56% (triangles), 75% (white circles), and 85% (white squares). Mean ± SD of three biological replicates for each measurement.

Figure 1 shows the germination of Col-0, *mtr4-1*, and *cat2-1* seeds stored in various combinations of temperature and RHs for more than 1 year (63 weeks) after their harvest. Germination was evaluated after 7 d at 25 °C in darkness, conditions in which *Arabidopsis* seed dormancy can be expressed (Leymarie *et al.*, 2012). At harvest, seeds from Col-0 were deeply dormant since their germination did not exceed 20%. Seeds of *mtr4-1* were more dormant than those from Col-0 since only 5% germinated, while *cat2-1* seeds were less dormant (75% germination; Fig. 1; Supplementary Fig. S1). Storage of seeds first resulted in a progressive increase of germination at 25 °C for all genotypes. In all cases, maximum germination percentage was reached after ~7 weeks of storage but the efficiency of dormancy alleviation greatly depended on temperature and RH. When temperature of storage increased from 10 to 25 °C, the optimal RH for dormancy alleviation increased from 33 to 75%, whereas the lowest RH (1%) prevented full dormancy release at all temperatures. Conversely, high RH (85%) also inhibited break of dormancy when temperature was lower than 25 °C.

Prolonged storage of seeds was associated with a dramatic decrease of germinability at 25 °C, which occurred after 9 weeks of storage at 10, 15, and 25 °C and did not depend on RH. At 20 °C, however, germinability decreased more slowly when seeds were stored at 56% RH and it became null only after ~50 weeks. This RH also extended germinability after storage at 15 °C to ~12 weeks (Fig. 1).

**Fig. 2.**
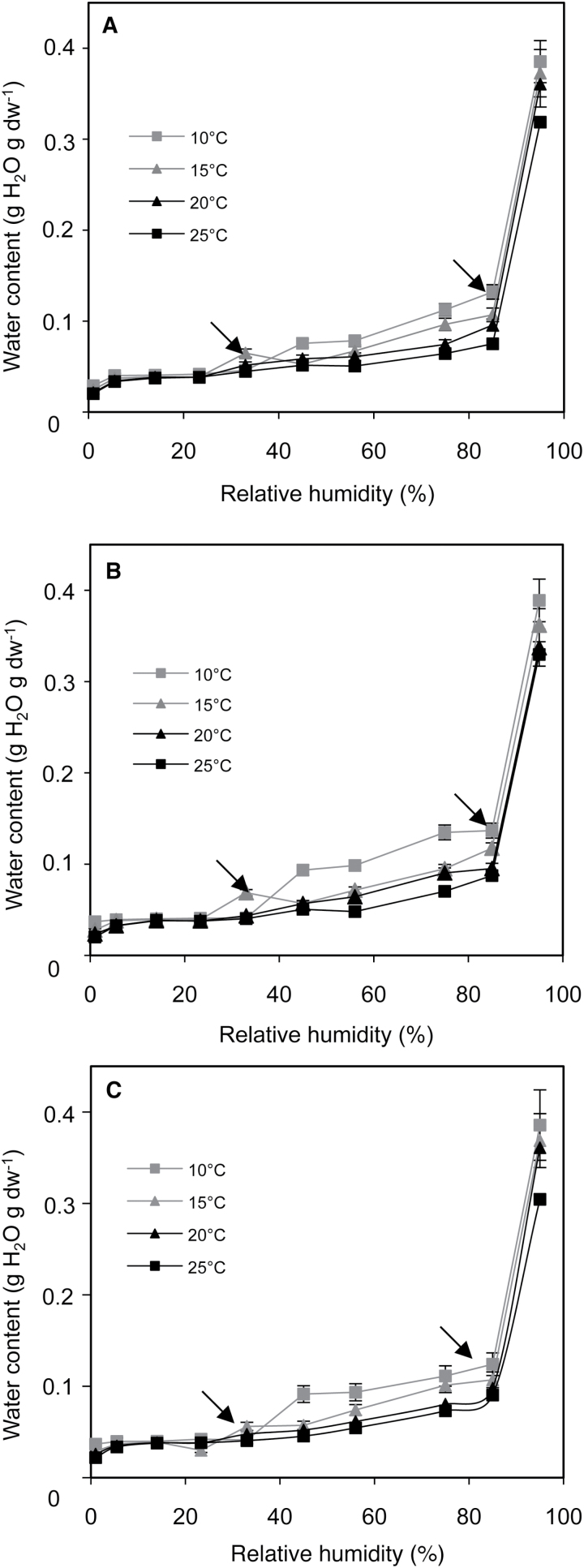
Water sorption isotherms measured at 10, 15, 20, and 25 °C of seeds of Col-0 (A), *mtr4-1* (B), and *cat2-1* (C). Arrows within graphs indicate limits of regions of water binding. Mean ± SD of 10 replicates of 10 seeds.

The relationship between RH and seed MC at different temperatures is given by the water sorption isotherms shown in Fig. 2. Whatever the genotype, changes in seed MC as a function of RH displayed the classical reverse sigmoidal curves of sorption isotherm allowing identification of three regions of water binding within seed tissues, as shown by arrows on the graphs. They correspond to bound water, which includes structural and monolayer water; intermediate water, where water is less firmly bound than in the first region; and free water. Above 85% RH, seed MC dramatically increased whereas it decreased at lower RHs (i.e. below ~15% RH) to reach MCs as low as 0.037g H_2_O g dw^–1^. RHs delimitating regions of isotherms were similar at all temperatures and did not vary for the mutants. As expected, decreasing temperatures were associated with higher water-binding capacities, particularly within the range 40–85% RH. For example at 56% RH, seed MC almost doubled when temperature decreased from 25 to 10 °C.

**Fig. 3.**
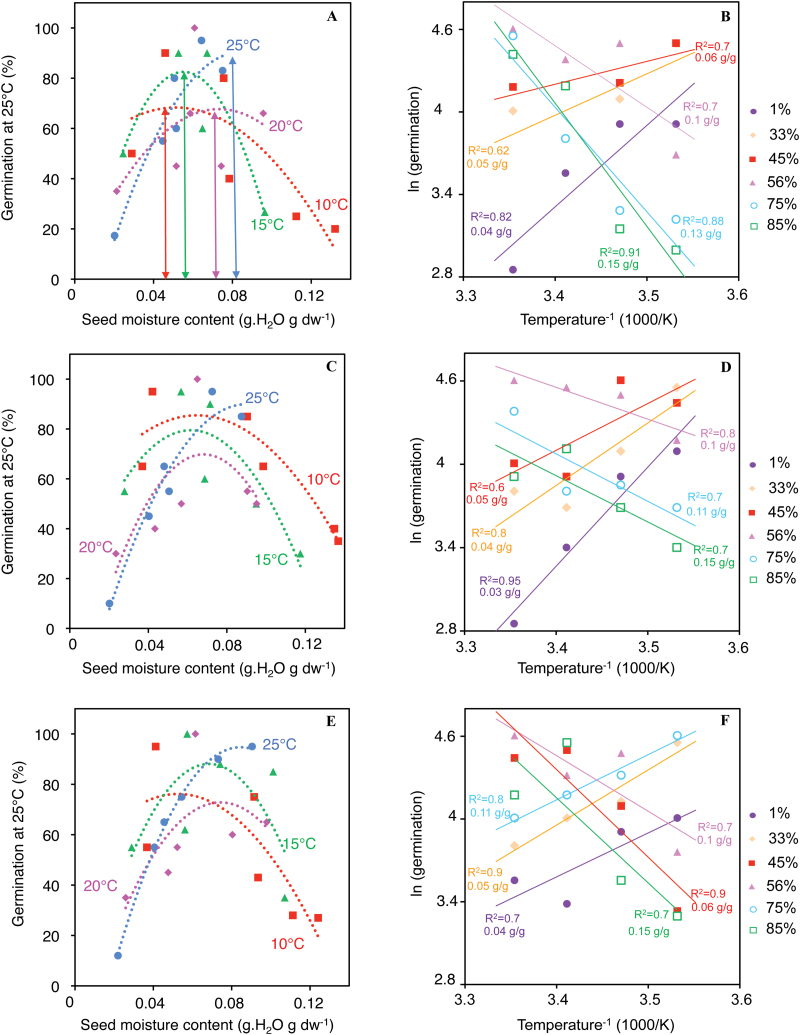
Germination after 7 d at 25 °C of Col-0 (A), *mtr4-1* (C), and *cat2-1* (E) seeds stored for 7 weeks at 10 °C (circles), 15 °C (diamonds), 20 °C (triangles), and 25 °C (squares) at various RHs (1, 33, 45, 56, 75, and 85%) which produced the indicated MC. Arrows in (A) indicate the optimal MC for dormancy release at each temperature. Arrhenius plots of seed dormancy release, where ln(germination), determined after 7 d at 25 °C, is plotted against the inverse of the temperature [Temperature^–1^ (1000/K)] for Col-0 (B), *mtr4-1* (D), and *cat2-1* (F). Seed MC during after-ripening (which lasted for 7 weeks) and *R* ^2^ values of the linear regression lines are indicated within the graph. (A colour version of this figure is available at *JXB* online.)

The optimal MC for seed dormancy release was determined at 10, 15, 20, and 25 °C (Fig. 3), which was evaluated by germinating seeds at 25 °C after 7 weeks of after-ripening, this duration being the most efficient for dormancy release (see Fig. 1). These results show a dependency of this value on temperature. The higher the seed MC, the higher the optimal temperature allowing breaking of dormancy was (Fig. 3A, C, E), and reciprocally. For example at 25 °C, the optimal MC for dormancy release of Col-0 seeds was close to 0.07g H_2_O g dw^–1^, but it decreased to 0.06 at 20 °C, 0.05 at 15 °C, and 0.04 at 10 °C as shown by arrows in Fig. 3A. Relatively similar values were obtained for seeds of *mtr4-1* and *cat2-1* mutants (Fig. 3C, D, respectively). One therefore can assume that the optimal MC for seed dormancy alleviation increased by approximately 0.02g H_2_O g dw^–1^ when temperature increased by 5 °C (Fig. 3A, C, E).

Arrhenius plots allow the temperature dependence of dormancy alleviation rate on RH to be determined (Fig. 3B, D, F). The relationship among seed MC, temperature, and dormancy alleviation is shown by the straight lines obtained when plotting ln(germination) against reciprocal temperature (1/T) and by the high correlation coefficients (Fig. 3B, D, F). The temperature dependence for dormancy release was similar for all genotypes (Fig. 3B, D, F). Below ~0.06g H_2_O g dw^–1^, the activation energy, determined by the slope of the plots, was negative (slope=–*E*a/*R*), i.e. the rate of dormancy alleviation increased when temperature decreased (Fig. 3B, D, F). Above this value, the activation energy became positive and increased when MC increased, i.e. the higher the seed MC, the higher the slope of the curves (Fig. 3B, D, F).

The temperature coefficient (Q10) allows measuring the rate of change of a biological system when increasing the temperature by 10 °C. The coefficient Q10 was calculated at different seed MCs within the range 10–25 °C (Table 1). It increased regularly from ~0.3 to >2 when seed MC increased from 0.04 to 0.15g H_2_O g dw^–1^ (Table 1), which indicated that rate of seed dormancy alleviation decreased when temperature increased at MC lower than 0.06g H_2_O g dw^–1^, then did not vary at intermediate MCs, i.e. when Q10 value was close to 1 and increased for higher MC. Q10 values ≥2, obtained when seed MC was higher than 0.13, indicated that rate of dormancy alleviation doubled when temperature increased by 10 °C.

**Table 1.** *Temperature coefficients (Q*_*10*_ *) of Col-0,* mtr4-1*, and* cat2-1 *seeds, calculated between 10 and 25 °C at different MCs*

Seeds	Values of Q_10_ at the seed MC (g H_2_O g dw^–1^)					
	0.04	0.05	0.06	0.1	0.13	0.15
Col-0	0.49	0.71	0.82	1.58	2.4	2.55
*mtr4-1*	0.31	0.61	0.75	1.2	1.96	2.14
*cat2-1*	0.36	0.69	0.9	1.44	2.16	2.29

### Physiology of seed germination after prolonged storage

**Fig. 4.**
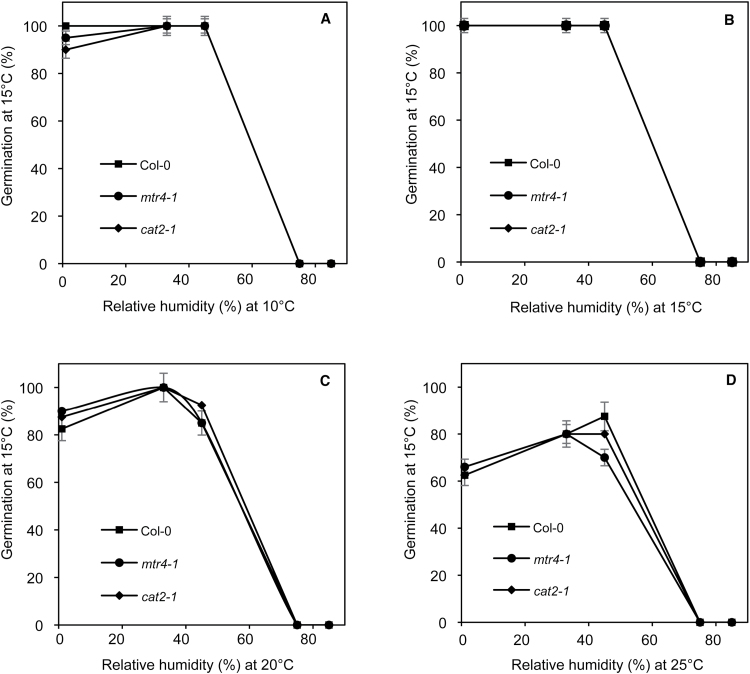
Germination after 7 d at 15 °C of Col-0, *mtr4-1*, and *cat2-1* seeds stored for 63 weeks at 10 °C (A), 15 °C (B), 20 °C (C), and 25 °C (D) under different RHs. Mean ± SD of three biological replicates for each measurement.

Since after 63 weeks of storage in all conditions germination percentages at 25 °C were always very low or null (Fig. 1), whether seeds stored for this duration were dead or deeply dormant was investigated. Germination at 15 °C, the optimal temperature for *Arabidopsis* seeds, revealed that seeds stored at 75% and 85% RH were dead after prolonged storage at all temperatures (Fig. 4), which was also confirmed by the absence of staining in the presence of tetrazolium chloride (TZ) (Table 2). Lower RHs maintained seed viability when seeds were stored at 10 or 15 °C, since seeds all germinated at 15 °C and almost all developed red staining in the presence of TZ (Table 2). This revealed a process of secondary dormancy induction. In contrast, increasing temperature of storage to 20 and 25 °C led to a decrease in seed viability, as shown Fig. 4, and by lower staining of tissues by TZ (Table 2). At these temperatures, only RHs close to 50% allowed seeds survival (Fig. 4 and Table 2). The use of several methods known to alleviate dormancy, such as cold stratification, GA, and ethylene treatments permitted seeds stored for 63 weeks to germinate at 25 °C (Table 2) and confirmed the data shown in Fig. 4.

**Table 2.** Percentages of germination and viability of Col-0 seeds stored for 63 weeks at various RHs at 10 °C, 15 °C, 20 °C, and 25 °C Germination assays were carried out at 25 °C for 7 d on gibberellic acid solution (GA_3_ 10^–4^ M), on water in the presence of ethylene (200 ppm), or after a stratification treatment of seeds at 4 °C for 2 d. The viability of seeds was assessed using tetrazolium chloride. Means ± SD of triplicate experiments.

**Storage temperature**	**Assay**	**Germination conditions**	**RHs (%**)					
			1	33	45	56	75	85
10 °C	Germination (%)	GA_3_	87.5±2.3	91±1.5	87.5±2.4	85±2.1	0	0
		Ethylene	85.7±1.7	92±1.4	86±1.2	86.5±1.4	0	0
		Stratification	86.5±1.8	90.5±2.8	87±2.4	87.4±1.7	0	0
	Viability (%)	-	86.5±0.6	90±1.2	85.6±1.4	85.6±0.8	0	0
15 °C	Germination (%)	GA_3_	82.5±2.5	81	85	82.5±2.5	0	0
		Ethylene	82.5±1.4	82	84	83.6±1.1	0	0
		Stratification	81.5±1.7	82.5±2.4	83.5	82.4±1.2	0	0
	Viability (%)	-	83.2±1.4	83.2±1.5	84±0.7	83.5±0.5	0	0
20 °C	Germination (%)	GA_3_	83.5±3.5	91	85	70	0	0
		Ethylene	85±3.5	89	84±1.2	70±1.2	0	0
		Stratification	84.5±3.7	88.5±2.4	85	73	0	0
	Viability (%)	-	83.2±1.3	90±0.7	84.5±0.8	71±1.3	0	0
25 °C	Germination (%)	GA_3_	69.5±3.2	88.5±3.5	81.5±4.1	82.5±3.1	0	0
		Ethylene	70.0±2.5	87.2±2.1	82.5±3.2	80±1.2	0	0
		Stratification	71.5±1.7	89.5±3.4	83.5±3.4	82.5±2.4	0	0
	Viability (%)	-	72±0.7	90±0.7	82±1.8	81±1.2	0	0

**Fig. 5.**
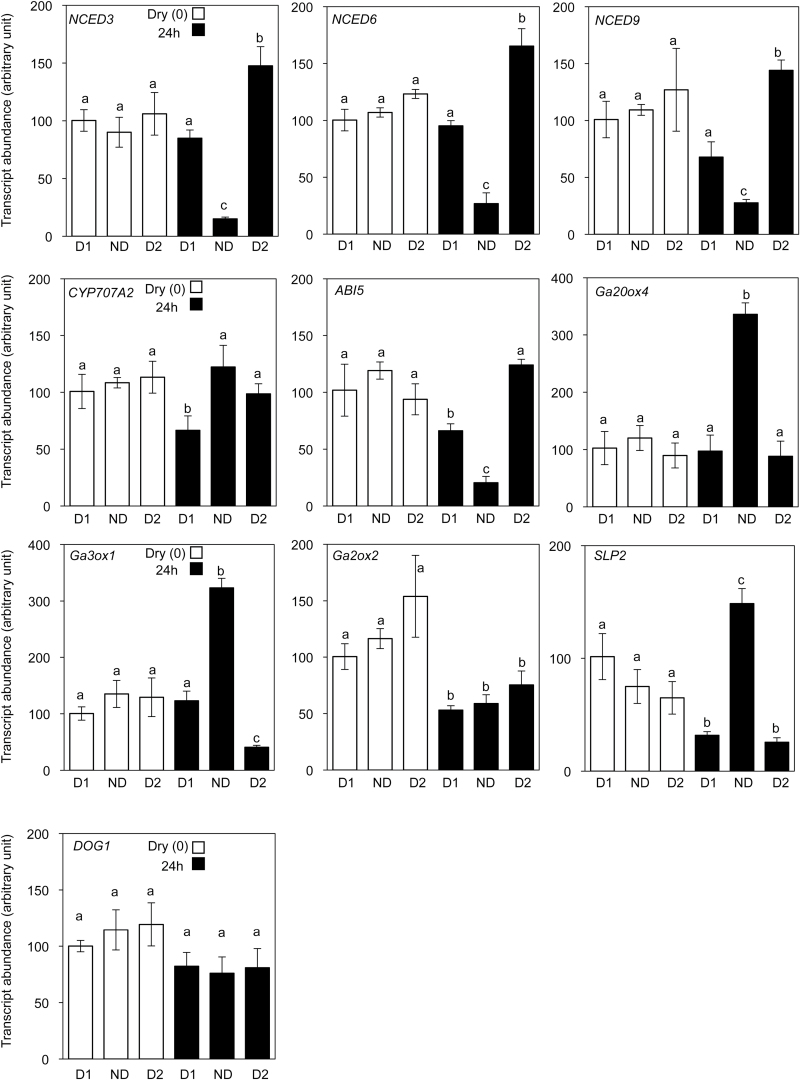
Transcript abundance of *NCED3*, *NCED6*, *NCED9*, *ABI5*, *DOG1*, *CYP707A2*, *Ga3ox1*, *Ga20ox4*, and *Ga2ox2* in Col-0 seeds, dry (white bars) or imbibed for 24h at 25 °C (black bars). Analysis was performed on dormant seeds after harvest (D1), non-dormant seeds after-ripened at 56% RH and 20 °C for 7 weeks (ND), and secondary dormant seeds stored for 63 weeks at 56% RH and 20 °C (D2). The relative expression was calculated from qRT-PCR data with two references genes, *Ubiquitin 5* and AT4G12590, and expressed in arbitrary units with a value of 100 attributed to the dry dormant seeds after harvest. Means ± SD of three biological replicates are shown. a, b and c indicate homogeneous groups in a corresponding class (Anova test and Newman–Keuls tests, *P*=0.05).

To gain more insights about the changes in dormancy level, the expression of genes known to be involved in the regulation of primary and secondary dormancy were investigated in freshly harvested seeds (D1), and seeds stored for 7 (ND) and 63 (D2) weeks at 56% RH and 20 °C. Gene expression was evaluated in dry seeds and in seeds imbibed for 24h at 25 °C in the dark, using real-time reverse transcription-PCR (RT-PCR) (Supplementary Table S1, available at *JXB* online). The abundance of all genes studied did not vary significantly when assessed in dry seeds (Fig. 5). The expression of *NCED3*, *NCED6*, *NCED9*, and *ABI5* followed the same pattern. It decreased significantly in non-dormant imbibed seeds (i.e. seeds after-ripened for 7 weeks) but increased markedly in seeds imbibed after prolonged storage (i.e. 63 weeks). In contrast, abundance of *CYP707A2*, *Ga20ox4*, *Ga3ox1*, and *SLP2* transcripts in imbibed seeds was higher in seed that had been after-ripened for 7 weeks than in freshly harvested seeds. After 63 weeks of storage, expression of these genes in imbibed seeds decreased to the same level as that found in freshly harvested seeds, excepted for *CYP707A2* whose expression did not decrease significantly. Finally, expression of *Ga2ox2* was similar in all imbibed samples. Abundance of *DOG1* transcript was not affected either by seed storage or by subsequent imbibition in darkness at 25 °C (Fig. 5).

## Discussion

At harvest, seeds of *Arabidopsis thaliana* were dormant since they were unable to fully germinate at 25 °C in darkness (Supplementary Fig. S1). Germination of seeds of the wild type Col-0 did not exceed 20% under these conditions. Freshly harvested seeds of the mutant *cat2-1* were less dormant since they germinated to 70% at 25 °C whereas seeds of the mutant *mtr4-1* displayed higher dormancy, their germination being lower than 10% at harvest. After-ripening carried out by placing dormant seeds at 56% RH and 20 °C induced dormancy alleviation and seeds of the three genotypes became able to fully germinate at 25 °C after 4 weeks under these conditions (Supplementary Fig. S1). *Arabidopsis* seed dormancy release by after-ripening is a classic mechanism but the consequences of modifying RH and temperature during this period are largely unknown. Here it is demonstrated that there exists a strong dependency of the physiological changes occurring in the dry state on both RH and temperature. It is also shown that the studied genotypes, and the level of dormancy at harvest, do not influence the pattern of fluctuation of dormancy level during storage. Therefore, the results obtained will be discussed below without making distinction among the wild type, Col-0, and the two mutants, *cat2-1* and *mtr4-1*.

### Primary dormancy alleviation dependency on temperature and RH

Studying the role of storage conditions of primary dormant *Arabidopsis* seeds revealed the complex relationship between seed MC and temperature for the control of dormancy release (Fig. 1). Whatever the genotype and the conditions of storage used here, total or partial dormancy release occurred within 7 weeks, but its kinetics greatly depended on RH and temperature. As already demonstrated (see Finch-Savage and Leubner-Metzger, 2006), it was shown here that, dormancy alleviation is favoured at intermediate RHs, i.e. close to 50%, but that it is slowed for extreme RHs, i.e. in very dried seeds or in high air humidity. It is also shown that dormancy release is faster at 20 °C, which allows proposing that the optimal combination for alleviation of dormancy in *Arabidopsis* seeds is 20 °C and 56% RH. From the sorption isotherms shown in Fig. 2, one can determine the corresponding optimal MC, which is close to 0.05g H_2_O g dw^–1^ at 20 °C (corresponding to 56% RH). This MC fell within the water-binding region 2 of sorption isotherms, which corresponds to weakly bound water but nevertheless limits biochemical reactions. Water sorption isotherms shown here displayed the classical sigmoidal shapes and the RHs at which the transitions occurred from different water-binding regions were roughly similar to those found in other ecotypes of *Arabidopsis* (Hay *et al.*, 2003) or in oily seeds such as sunflower (Bazin *et al.*, 2011).

The results presented here reveal a tight and complex relationship between temperature and RH in the kinetics of after-ripening, and Fig. 3 demonstrates that, the higher the seed MC, the higher the optimal temperature for after-ripening. For example, similar kinetics of seed dormancy alleviation can be obtained at 10 °C or 25 °C if seed MC is close to 0.04 or 0.08g H_2_O g dw^–1^, respectively. Thermodynamic components of seed dormancy release can be appreciated using Arrhenius plots (Fig. 3B, D, F) and calculation of Q10 values (Table 1). The effect of temperature on enzyme activities is well described by the Arrhenius activation energy, which represents the effect of temperature on the catalytic rate constant, and it is widely admitted that rates of biological activity increase exponentially with temperature (Brown *et al.*, 2004). However, dry seeds are a case species in which absence of free water prevents active metabolism. Arrhenius plots shown in Fig. 3B, D, F allow proposing the seed MC value of 0.06g H_2_O g dw^–1^ as being critical for governing the nature of the mechanisms leading to seed dormancy release. Indeed, below 0.06g H_2_O g dw^–1^ dormancy release in *Arabidopsis* seed was associated with negative activation energies, as shown by the negative slot of the plots (Fig. 3B, D, F). The rate of dormancy release thus decreased with increasing temperature. In sunflower seeds, it was also found that dormancy alleviation during after-ripening was associated with negative activation energies, when seed MC was below 0.1g H_2_O g dw^–1^ (Bazin *et al.*, 2011). For biological models, negative activation energies have been shown in mammalian cell lines exposed to hypothermia, which causes chilling injury and killing and they have been attributed to gel-to-liquid crystalline transition of lipids and protein denaturation (Muench *et al.*, 1996). However, in the present study, the major determinant was MC, but not temperature, since negative and positive activation energy occurred within the range 10/25 °C, as also shown by the Q10 values, which are clearly below 1 at lowest MC (Table 1). In plants, metabolic rates double with every 10 °C increase in temperature usually giving Q10 values close to 2 (Criddle *et al.*, 1994). This was the case here but only when seed MC was higher than 0.12g H_2_O g dw^–1^ (Table 1). Altogether these data therefore suggest that dormancy alleviation at low MC does not involve metabolic activities, contrary to what occurs when seed MC increases and when Q10 values are close to 2 (Table 1). As suggested for dormancy alleviation of sunflower seeds, dormancy release of *Arabidopsis* may be achieved by distinct mechanisms as a function of seed MC. At low MC, it is proposed that seed dormancy release occurs through non-enzymatic oxidative reactions, which are known to be associated with negative activation energies (Cheney, 1996; Cunningham *et al.*, 1999; Olson *et al.*, 2002; Bazin *et al.*, 2011). Elementary reactions exhibiting negative activation energies are reactions for which increasing the temperature reduces probability of the colliding molecules capturing one another. This is in agreement with the concept of an ‘oxidative window’, which postulates that a certain oxidative load is needed to break dormancy (Bailly *et al.*, 2008). When seed MC is above ~0.12g H_2_O g dw^–1^, metabolic reactions are likely to occur and to participate in dormancy alleviation. Then, the activation energy (given by the slope of the plots) increases when seed MC increases thus showing that changes in temperature have a significant effect on the rate of dormancy alleviation. Finally, between 0.06 and 0.12g H_2_O g dw^–1^, the activation energy is low within the studied temperature range, which shows that changes in temperature have then little effect on the rate of dormancy alleviation.

### Loss of seed germinability during prolonged storage

Following partial or full dormancy release during the first weeks of after-ripening, seed germination at 25 °C abruptly decreased and became very low after 12 weeks of storage (Fig. 1). Nevertheless, storing seeds for longer periods induced again an increasing ability to germinate at this temperature, and maximum germination percentages were reached after 21–28 weeks of storage, even though they never exceeded 35%. Interestingly, the combination of RHs and temperatures, which were the more efficient for triggering release of primary dormancy (see Fig. 3), did not permit a marked increase of germination after 21–28 weeks of storage (Fig. 1). Finally, the ability to germinate at 25 °C declined again and was fully prevented after 1 year of storage, whatever the conditions of storage. These fluctuating changes in the germination potential during dry storage were rather unexpected. Indeed, it is very well known that seed germinability decreases during storage as a result of ageing (Rajjou and Debeaujon, 2008) but what is shown here is a re-induction of the potential to germinate during storage after an initial loss of germinability. This suggests that loss of germinability after 12 weeks of storage did not result from a loss of viability but from an induction of secondary dormancy. This hypothesis was verified by germinating seeds stored for 63 weeks, i.e. whose germination was totally prevented at 25 °C, at lower temperature (Fig. 4) or at 25 °C in the presence of ethylene or GA, or after stratification at 5 °C (Table 2). The high germination rates observed under these conditions show that stored seeds were fully viable, as also observed with TZ staining, but that they were unable to germinate at 25 °C, provided that RH during storage did not exceed 56% (Table 2). In this former case, seeds were no more viable as shown by the absence of TZ staining or by the inefficiency of dormancy release treatments (Table 2).

These data therefore allow the identification of two different mechanisms occurring during prolonged storage of *Arabidopsis* seed. First, the decline of seed viability at high RH (>56%) (Table 2) was a consequence of seed ageing. Seed ageing was also favoured at 20 and 25 °C at the lowest RH (Fig. 4), which might result from increased lipid oxidations, which are known to be enhanced within region 1 of sorption isotherm and with increasing temperature (Labuza, 1980). Interestingly, there was no correlation between seed sensitivity to ageing during the storage period studied here and the initial level of seed dormancy (Supplementary Fig. S1), contrary to what was shown by Nguyen *et al.* (2012). Second, it was demonstrated that secondary dormancy can be induced at low MC. While primary dormancy is set during seed development, secondary dormancy develops after harvest when environmental conditions do not allow germination (Hilhorst, 2007). In natural conditions, when seeds are buried in the soil, light, temperature, and MC, through their natural seasonal variation, are the key factors regulating dormancy cycling (Baskin and Baskin, 1998; Footitt *et al.*, 2011). In the experiments described herein, these factors were maintained at constant levels throughout storage and changes in dormancy level thus resulted from intrinsic properties. This was also rather surprising that all seed batches, i.e. stored in various conditions or issued for different genotypes, displayed synchronized and similar fluctuations in dormancy level. A relatively similar behaviour has been evidenced for seeds of *Mesembryanthemum nodiflorum* L. stored in dried conditions (Gutterman and Gendler, 2005), which displayed an annual rhythm of germination. Seasonal or circadian rhythms are well described in plants but they generally concern metabolically active organisms (see McClung, 2006). The mechanisms underlying fluctuating changes in dormancy level in the dry state during storage are totally unknown and difficult to explain with regard to the actual knowledge related to life rhythms.

Gaining insights in the changes of dormancy level during storage was achieved by studying the expression of key regulator genes of dormancy. It was shown that seed dormancy cycling in the dry state relied on quantitative changes during seed imbibition of transcripts related to hormone synthesis and signalling. As expected, abundance of the transcripts studied did not vary during long-term seed storage (1 year) at 20 °C and 56% RH (Fig. 5), a condition that retained seed viability but that induced secondary dormancy (Table 2 and Fig. 4). Indeed at this RH, seed MC was close to 0.05g H_2_O g dw^–1^ thus preventing transcription (Meimoun *et al.*, 2014). However, seed imbibition at 25 °C for 24h revealed major changes in gene expression, as regulated by dormancy status. Release of primary dormancy, i.e. after 7 weeks of storage, was associated with the repression of genes related to ABA biosynthesis *(NCED3*, *NCED6*, *NCED9*) and signalling (*ABI5*) and with the induction of *CYP707A2*, a gene involved in ABA catabolism (Fig. 5). Simultaneously, expression of *GA20ox4* and *GA3ox1*, both involved in GA activation (Yamaguchi, 2008), increased concomitantly with that of *SLP2*, a gene known to be induced by GA (Ogawa *et al.*, 2003). This shows that alleviation of primary dormancy is regulated by the hormonal balance of ABA/GA, as is already well known for seeds of this species (Finch-Savage and Leubner-Metzger, 2006). Interestingly, induction of secondary dormancy during subsequent storage globally restored the same pattern of gene expression, estimated after 24h of imbibition at 25 °C, as the one found in primary dormant seeds. It can be concluded that this similarity in both the primary and secondary dormant states imposes maintenance of dormancy through ABA synthesis and signalling, and repression of GA synthesis and signalling, thus inducing a negative regulation of germination during seed imbibition, as already postulated by various authors (e.g. Cadman *et al.*, 2006; Finch-Savage and Leubner-Metzger, 2006; Finch-Savage *et al.*, 2007; Footitt *et al.*, 2011). These results nevertheless demonstrate that the period of storage experienced by the seeds is critical for regulating the expression of regulators during subsequent imbibition. In particular, it was demonstrated that a prolonged period of dry storage leads to fluctuating variation of transcript abundance during subsequent seed imbibition. However, how storage in low RH can influence the ability of seeds to regulate expression of specific genes during their imbibition is largely unknown. Finally, it has been shown that the abundance of *DOG1* transcript remained unchanged in all the conditions studied. This is rather surprising since primary and secondary dormancy have previously been associated with the accumulation of *DOG1* (Bentsink *et al.*, 2006; Footitt *et al.*, 2015). However, Leymarie *et al.* (2012) have also shown that *DOG1* did not accumulate differentially during imbibition of dormant and non-dormant seeds of Columbia at 25 °C in darkness, which raises the question of the regulation of *DOG1* expression by light.

In conclusion, this study brings a comprehensive view of the combined effects of temperature and RH on the physiology of *Arabidopsis* seeds during prolonged storage. From a practical point of view, these results should lead scientists interested by studies on dormancy using the model plant *Arabidopsis* to pay attention to the conditions of storage used in their experiments. Besides revealing the effects of various combinations of temperature and RH on the kinetics of seed dormancy release and ageing, this work highlights cyclic and intrinsic fluctuations in dormancy, which do not depend on temperature and MC, contrary to release of primary dormancy. In nature, this fluctuation might help seeds to determine the temporal window for germination and might constitute a temporal signal for determining the time after shedding, i.e. the time of the year, independently of spatial signals for germination, such as light or temperature, which cannot be sensed by dry seeds. This mechanism could be of a particular importance after drought periods, i.e. when water limitation does not allow seed hydration. In this case, the low seed MC probably prevents their perception of the seasonal timing for germination, but this failure could be fixed by the intrinsic fluctuation of dormancy. At last, this work also raises fundamental questions about the molecular bases of the changes that can take place in the dry state, but they will require further investigations.

## Supplementary data

Supplementary data are available at *JXB* online.

Supplementary Fig. S1. Germination at 25 °C in darkness of Col-0, *mtr4-1*, and *cat2-1* seeds.

Supplementary Table S1. Sequences of primers used for qRT-PCR experiments.

Supplementary Data
