# Supplementary material for: Fluctuation of Arabidopsis seed dormancy with relative humidity and temperature during dry storage
Source: J Exp Bot. 2015 Oct 1;67(1):119–30. doi: 10.1093/jxb/erv439 (PMC4682427; doi:10.1093/jxb/erv439)
Supplement: Supplementary Data [file supp_67_1_119__index.html]

Fluctuation of Arabidopsis seed dormancy with relative humidity and temperature during dry storage — Fluctuation of Arabidopsis seed dormancy with relative humidity and temperature during dry storage — Supplementary Data 

# Fluctuation of Arabidopsis seed dormancy with relative humidity and temperature during dry storage

## Supplementary Data

Data files

- Supplementary Data - Supplementary Data
- Supplementary Data - Supplementary Data
