# Supplementary material for: Fluctuation of Arabidopsis seed dormancy with relative humidity and temperature during dry storage
Source: J Exp Bot. 2015 Oct 1;67(1):119–30. doi: 10.1093/jxb/erv439 (PMC4682427; doi:10.1093/jxb/erv439)
Supplement: Supplementary Data [file supp_erv439_jexbot152595_file002.pdf]

Supplemental Table S1. Sequences of primers used for qRT-PCR experiments. The AGI for each gene were determined by Tair and the sequence of primer by Primers 3.

Genes	AGI	Primer sequence
ABI5	AT1G18480	**F**: TTGAAGTCAAGGGCACAACC<br>**R**: CGGGTTCCTCATCAATGTCC
CYP707A2	AT2G29090	**F**:GGCACCAAAACCTTACACG<br>**R**:TCTCCAATCACTTCCCATCTG
DOG1	AT5G45830	**F**: GCTCTAATTTGGATGGGTGGT<br>**R**: GTCGATGTTGCGGAGAAACT
Ga3ox1	AT1G18250	**F**: TTGGGGTCAGCGAAGAAGA<br>**R**:CAGAATGGTTAGGAGGGTGGA
Ga20ox4	AT5G64100	**F**: GCGAGACGACAAGGAAGACA<br>**R**: TCGGGATACGCTCTCTCACC
Ga2ox2	AT1G74670	**F**: TGTTGGAGATGGTTGCCGAA<br>**R**: CCATCTTCTCCGCCTCTTCC
NCED3	AT1G72100	**F**: GCTGCGGTTTCTGGGAGAT<br>**R**:GGCGGGAGAGTTTGATGATT
NCED6	AT3G51600	**F**: TTCAAGATACCGACACTTCCTG<br>**R**: GGCGATTCTGCTCCATAGG
NCED9	AT1G69530	**F**: TCCCCTGCTATGTTTCTTCC<br>**R**:AGACGGTGGTTTGAATGTCG
SLP2	AT4G34980	**F**: AAAATCGGGAATGTCATCAGC<br>**R**: CAACCAATCCTTTGGCTACG
