# Supplementary material for: Fluctuation of Arabidopsis seed dormancy with relative humidity and temperature during dry storage
Source: J Exp Bot. 2015 Oct 1;67(1):119–30. doi: 10.1093/jxb/erv439 (PMC4682427; doi:10.1093/jxb/erv439)
Supplement: Supplementary Data [file supp_erv439_jexbot152595_file001.pdf]

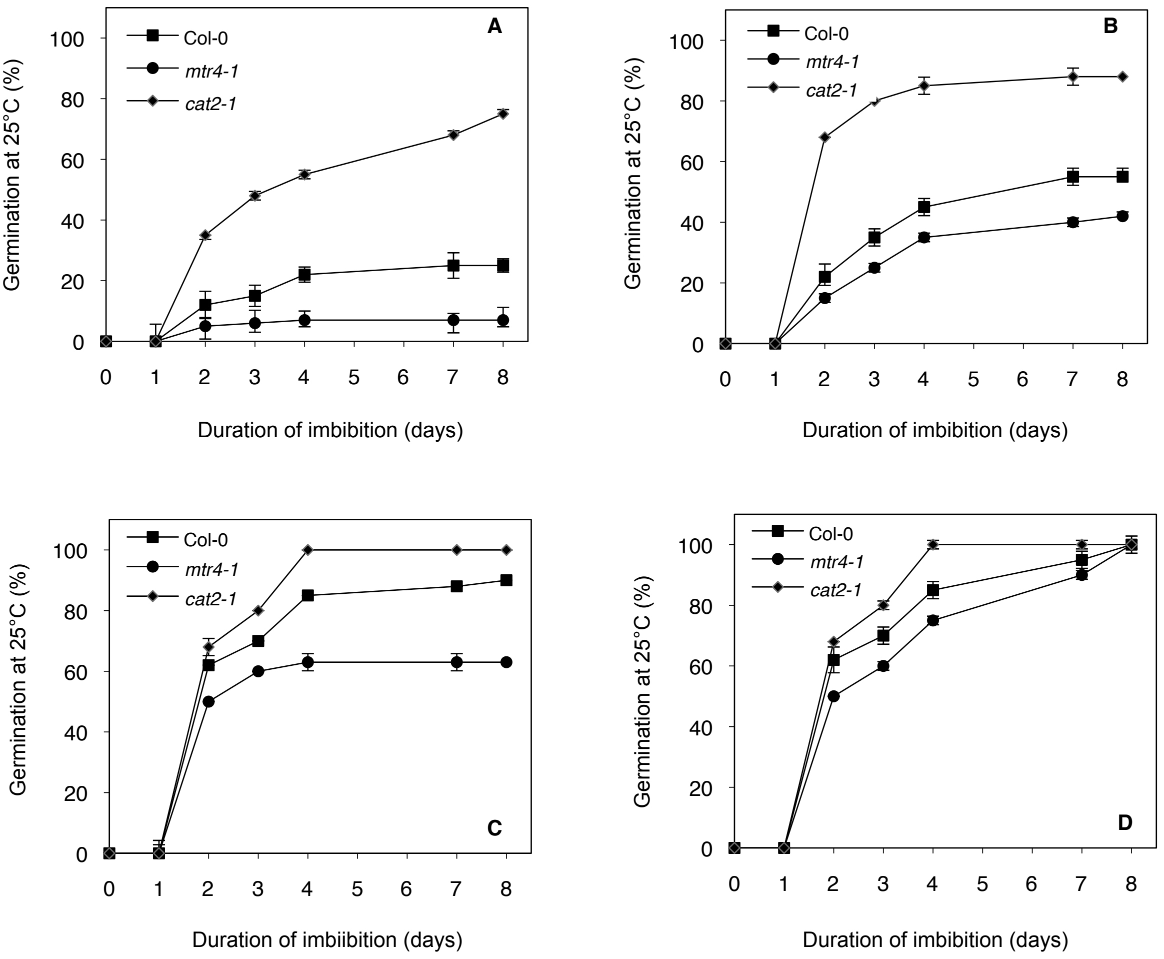

Supplementary Fig. S1. Germination at 25°C in darkness of Col-0, *mtr4-1* and *cat2-1* seeds after harvest (A), after storage at 56% relative humidity and 20°C for 2 (B), 3 (C) or 4 (D) weeks. Mean ± standard deviation of 3 biological replicates for each measurement.
